# Osteoprotegerin Induces CD34^+^ Differentiation in Endothelial Progenitor Cells

**DOI:** 10.3389/fmed.2018.00331

**Published:** 2018-11-27

**Authors:** Catherine Boisson-Vidal, Zahia Benslimane-Ahmim, Anna Lokajczyk, Dominique Heymann, David M. Smadja

**Affiliations:** ^1^Inserm, UMR_S1140, Faculty of Pharmacy, Université Paris Descartes, Sorbonne Paris Cité, Paris, France; ^2^Inserm, UMR_S1232, CRCINA, Institut de Cancérologie de l'Ouest, Université Nantes-Angers-Le Mans, Nantes, France; ^3^AP-HP, Hematology Department, European Georges Pompidou Hospital, Paris, France

**Keywords:** osteoprotegerin, endothelial progenitor cells, endothelial-colony forming cells, CD34^+^ cells, proliferation

## Abstract

Endothelial progenitor cells (EPCs) are the main hypothetical cells that could give rise to vessels and in particular one subtype isolated from peripheral or cord bloods: endothelial colony forming cells (ECFCs). These ECFCs are clonogenic precursors committed to endothelial lineage and have robust vasculogenic properties. However, their low number and poor expansion properties when isolated from human adult bloods, currently limit their use as an autologous cell therapy product. We previously reported that osteoprotegerin (OPG), a well-characterized regulator of bone metabolism, contributes to ischemic tissue revascularization, tumor growth *in vivo*, and potentiates ECFCs proangiogenic properties through the secretion of SDF-1. The current study investigated the role of OPG in ECFCs differentiation and expansion from cord blood CD34^+^ cells. OPG increased the number of ECFCs after endothelial differentiation of CD34^+^ cells, enhancing the time of EPCs colonies initial appearance and the growth kinetic of endothelial cell progeny. OPG-exposed ECFCs expressed higher levels of CD34^+^ compared to control ECFCs. In conclusion, our findings provide novel insights into OPG in regulation of CD34^+^ progenitor cells. These results give new opportunities for *ex vivo* expansion of human ECFCs using OPG as a cell culture component for future ECFC product manufacture according to GMP.

## Introduction

Endothelial progenitor cells (EPCs) are the main hypothetical cells that could give rise to vessels and in particular a specific subgroup of circulating EPCs isolated from cord and adult peripheral blood: Endothelial colony forming cells (ECFCs) (1). These highly proliferative non-hematopoietic phenotype ECFCs are precursors committed to endothelial lineage. They are clonogenic and have robust vasculogenic properties. The absence of any specific markers in contrast to mature cells limited the ability to identify them in early 2000. We now have convincing data showing a clear difference between EPCs with mature cells (2–5). Their low number and poor expansion properties when isolated from human blood or bone marrow currently limit their use as an autologous cell therapy product (6). Strategies to improve ECFCs therapeutic potential are needed and one of the challenges is to understand the main effectors allowing an endothelial differentiation from immature cells and/or circulating progenitors.

Osteoprotegerin (OPG), a soluble member of the tumor-necrosis-factor family, is a well-characterized regulator of bone metabolism, which acts by blocking osteoclast maturation. It plays key roles in regulating numerous other physiological and pathological processes especially in vascular system (7). OPG is constitutively secreted by endothelial cells and their progenitors, as well as by smooth muscle cells, megakaryocytes, and platelets (8, 9). It promotes growth and tubule formation of mature endothelial cells (10). We recently described an increase in ECFCs vasculogenic properties *in vitro* and *in vivo* (11, 12). These beneficial effects have been attributed at least in part to SDF-1/CXCR-4 and heparan proteoglycan pathways (13). OPG has also been described to induce hematopoietic stem cells expansion (14) and to mediate cardioprotection by protecting the cells from reactive oxygen species-induced cell death (15). Because commitment of stem/progenitor cells into ECFCs is a main limitation to their clinical use, we hypothesized that OPG might regulate biological ECFCs maturation and investigated the role for OPG in ECFCs differentiation from cord blood CD34^+^ cells.

## Materials and methods

### Buffy coat cell preparation and culture of umbilical cord blood endothelial cells

The study was approved both by the relevant ethics committee (Hôpital Saint Louis, Paris, France) and the French Ministry of Higher Education, Training and Scientific Research (AC-2008-376).

Mononuclear cells were isolated from human cord blood by density-gradient centrifugation on Pancoll and CD34^+^ mononuclear cells by magnetic-bead separation according to the manufacturer's instructions (Miltenyi Biotec, France). ECFCs were obtained and cultured with or without OPG (25 ng/mL) as previously described (5, 11). Colonies were identified by their characteristic morphology then by immunostaining for von Willebrand factor and double-positivity for DiI-AcLDL uptake and BS-1 lectin binding.

### Cell proliferation potential

ECFCs appeared as small compact cell clusters about 10 to 20 days after plating. Cells from each colony were replated on 12-well dishes then on 6-well dishes and finally in a T25 flask. Subsequently, confluent cells were replated in T25 flasks every 3–4 days until day 50. The trypan blue exclusion test was used to determine cell counts at each passage. These counts served to plot a growth kinetic curve and calculate the population doubling time (PDT) and cumulative population-doubling level (CPDL) as previously described (16).

### Immunophenotyping of endothelial cells

On culture days 25 and 40, ECFCs cultured with and without OPG were detached using accutase then incubated with primary or isotype control antibody and analyzed by fluorescence-activated cell sorting using a FACSCalibur cytometer (Becton Dickinson, France). We used directly conjugated primary murine monoclonal antibodies specific for the following surface antigens: CD34, CD144, CD105 (Beckman Coulter, Cylex, France); CD133 (Miltenyi Biotec, France); CD31, CD73, CD61/51, VEGF-R2 (BD Pharmingen, France); CD146 (Santa Cruz Biotechnology, France); CD54, CD106 (AbCys, France), CD45, and CD115 (Immunotech, France). Corresponding isotype stains were used as negative controls. Data plotting was performed using CellQuest software (BD Biosciences, France).

### Statistical analysis

Differences between groups were assessed using Student's paired *t*-test, with the statistical software package GraphPad Prism, version 5 (GraphPad Software, USA). *P* ≤ 0.05 were considered statistically significant.

## Results

### CD34^+^ cells cultured with OPG exhibits increased clonogenic capacity and proliferative potential

**Figure 1:**
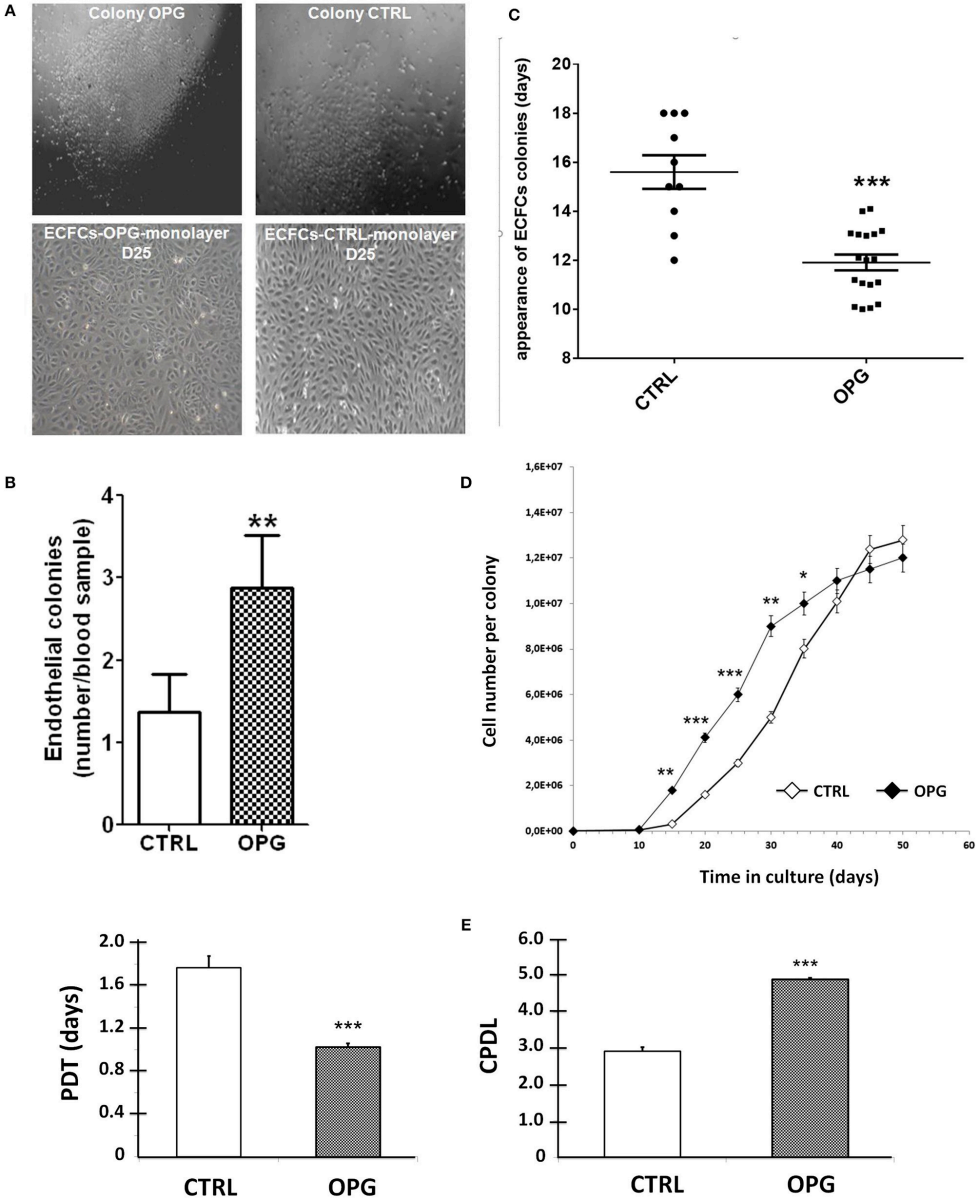
Osteoprotegerin (OPG) increases the colony-forming capacity and proliferative potential of EPC-derived endothelial colony-forming cells (ECFCs). Mononuclear cells isolated from cord blood were cultured in EGM2 with (OPG) or without (CTRL) 25 ng/ml of OPG. **(A)** Representative photomicrographs (10 × magnification) of endothelial progenitors-derived ECFCs from the OPG-exposed and control groups after 14- and 25-day of the endothelial cell progeny derived from the CD34^+^ EPC colonies grown to confluence. **(B)** Number of colonies per equivalent cord blood volume. The number of colonies formed was identified by phase-contrast microscopy. Results represent the mean ± SEM ECFCs of 8 independent experiments. **(C)** Time of initial ECFCs appearance after culture initiation from equivalent volumes of cord blood. Results represent the mean ± SEM number of days before initial ECFCs appearance of 10 independent experiments for CTRL conditions and 18 independent experiments for OPG conditions. **(D)** Growth kinetics of the endothelial cell progeny derived from cord EPC colonies. *n* = 10, cells were enumerated at each passage. **(E)** Population doubling time (PDT) and Cumulative Population-Doubling Level (CPDL) of EPCs-derived ECFCs during 15 days of culture. *P < 0.05, **P < 0.01 and ***P < 0.001 by Student paired *t*-test.

We firstly examined the possible involvement of OPG in cord blood CD34^+^ commitment to ECFCs. We harvested CD34^+^ cells from umbilical cord bloods and observed ECFCs formation in the presence or absence of 25 ng/ml of OPG added to the culture medium EGM2 from the first day of culture. We obtained colonies in both groups that displayed the same cell phenotype with a cobblestone morphology (Figure 1A). As shown in Figure 1B, OPG increased significantly the number of colonies (2.9 ± 0.6 vs. 1.4 ± 0.5 colonies per equivalent cord blood volume for OPG and control conditions, respectively, *p* = 0.0084) and decreased the timing of colony emergence (Figure 1C, *p* = 0.0008). The EPCs-derived colonies emerged 4 days earlier than in control culture medium. These observations suggested that OPG stimulated ECFCs formation.

We then compared the proliferative kinetics of ECFCs cultivated in the presence or absence of OPG from the very first day of CD34^+^ culture. As shown in Figure 1D, the growth curves indicate greater proliferative potential of ECFCs exposed to OPG compared to controls conditions: after a 6-day latency period, the number of cells in the OPG group showed a rapid increase to 9 × 10^6^ on day 30 followed by a slower increase. Control ECFCs also proliferated rapidly but less actively, so that 37 days were needed to obtain 9 × 10^6^ cells. The presence of OPG in the culture medium induces a 1.8-fold decrease in the average PDT and a 1.7-fold increase in the CPDL of ECFCs the first 15 days of culture (Figure 1E, *p* < 0.0001).

### OPG modulates CD34 surface expression by ECFCs

**Figure 2:**
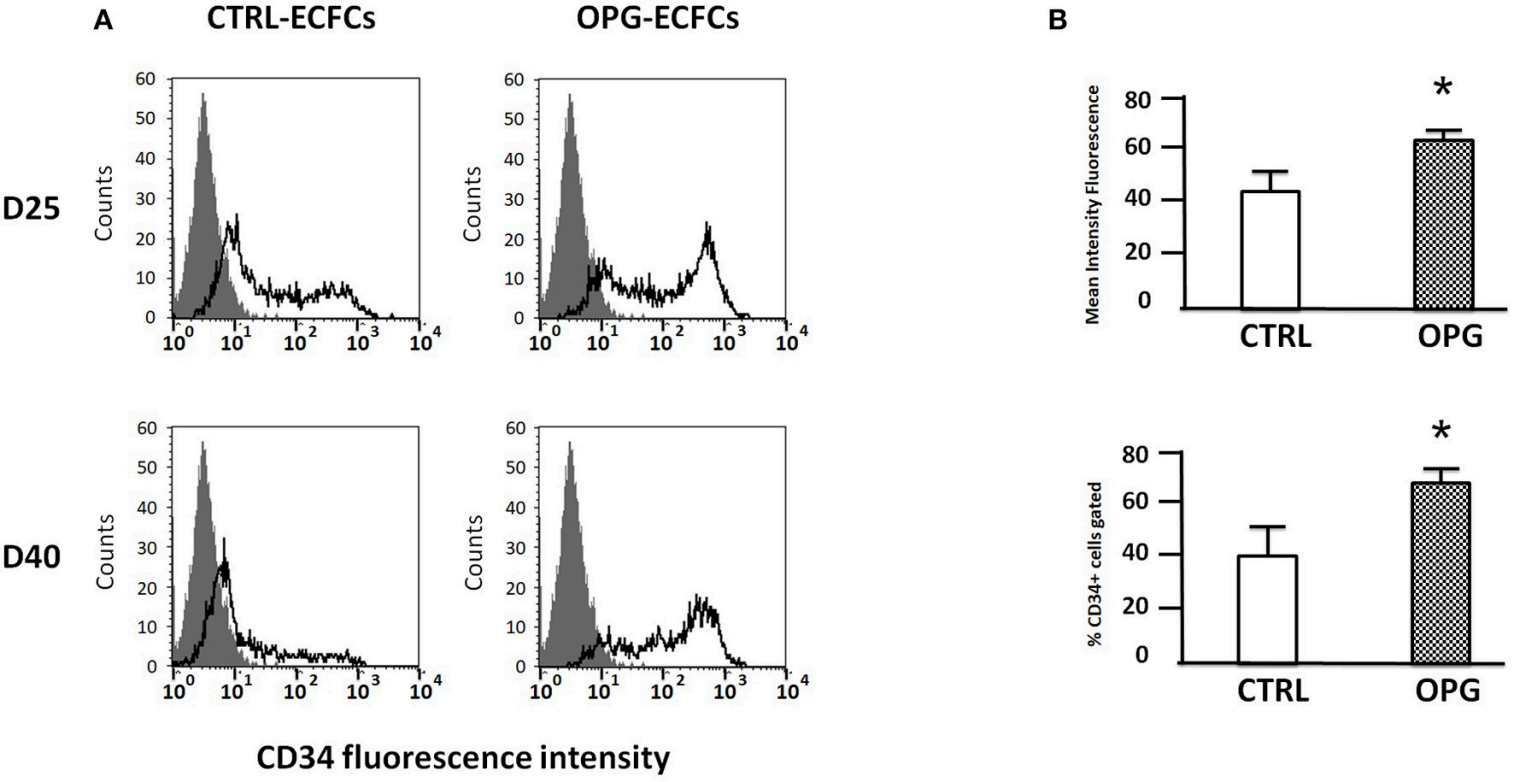
Comparative immunophenotyping of osteoprotegerin (OPG)-exposed and control endothelial colony-forming cells (ECFCs) after 25 and 40 days of culture. **(A)** Representative examples of CD34 expression profiles 25 and 40 days following the seeding. The isotopic control is in gray and the specific antibody in black. **(B)** Mean fluorescence intensity of OPG-exposed and control ECFCs CD34^+^ marker analyzed by flow cytometry. MFI ≥ 2 were considered positive (MFI ± SD, *n* = 4 for CTRL and *n* = 5 for OPG conditions, *P < 0.05 by Student paired *t*-test).

To investigate OPG influence on ECFCs phenotype, we used flow cytometry to monitor the expression of selected markers on days 25 and 40 in the OPG and control groups. Mature endothelial cells markers (vWF, P-selectin) were highly expressed (data not shown). Immunophenotyping revealed that ECFCs cultivated under both conditions express the endothelial cell-surface antigens CD31, CD105, CD144, CD146, and VEGR-R2 at approximatively the same level. None of the groups expresses any significant amount of hematopoietic cell surface antigen CD45, CD14, and CD115. However, we found on day 25 that OPG-exposed ECFCs expressed higher levels of CD34^+^ compared to control ECFCs (Figures 2A,B, *p* = 0.04). The percentage of CD34-positive cells was increased 1.6-fold for OPG-exposed ECFCs compared to control cells (Figure 2B *p* = 0.04). By day 40, the CD34 expression decreased in both groups but remained slightly higher in the OPG group (data not shown).

## Discussion

Our present study demonstrates the promoting effect of OPG on CD34^+^ differentiation in ECFCs which have then stronger capacity to proliferate. ECFCs cultured in the presence of OPG strongly expressed CD34^+^ and are devoid of hematopoietic markers (CD45, CD14) conforming that they are not originated from hematopoietic stem cell pool. This suggests that OPG could contribute to endothelial commitment and give rise to expanded CD34^+^-ECFCs with better vasculogenic properties. ECFCs exposed to OPG may display a higher proliferative ability allowing a faster generation of sufficient number of cells for clinical applications.

Current protocols for isolation and expansion of ECFCs relies on the presence of serum and various growth factors to promote endothelial cells proliferation and differentiation (16–20). Previous studies have shown that long term expansion reduces their proliferative and angiogenic potency (16, 21–24). We demonstrated here that exogenous OPG is able to increase endothelial differentiation from CD34^+^ stem cells and to give them enhanced proliferative potential. This further highlights the importance of OPG in progenitors related biology.

Few data describe the pathways that allow the endothelial differentiation of stem/progenitor cells (25). Several methods developed in the purpose of improving the isolation and long term expansion of ECFCs while preserving their angiogenic potential have shown that human platelet lysate (PL) are suitable serum substitutes (1, 20, 26, 27). This could be related at least in part to the presence of OPG contained in megakaryocytes and alpha-granules of platelets (8, 9). Our results could explain the efficacy of PL to yield twice more colonies per ml blood compared to the conventional isolation medium with fetal bovine serum (27).

### Regarding CD34^+^ cells survival and expansion

Numerous factors have been described to promote ECFCs proliferation and maturation in culture (TGF-beta, VEGF, BMP4, etc.) (6). We can explain the observed enhanced proliferative properties of ECFCs primed with OPG through different mechanisms. (i) OPG could increase directly or indirectly the proliferation of ECFCs. We previously observed that OPG increase SDF-1 expression and secretion in ECFCs (11). However, SDF-1 does not have any effect of ECFCs proliferation (28). Furthermore, OPG does not have any effect on RNA expression nor secretion of VEGF, the main effector known in ECFCs proliferation and maturation [data not shown, 29]. OPG could induce the secretion of specific growth factor(s) that could be involved in proliferation. OPG has an additive effect to VEGF, angiopoietin-1, TGFβ-1, and IL8 in PL, and may further support efficient outgrowth of ECFCs (30, 31). (ii) Effects of OPG on ECFCs proliferation could result from pro-apoptotic genes down-regulation already described (13). OPG protects HUVECs and ECFCs from apoptosis induced by growth factor deprivation and preserves their viability by stimulating mTOR and Akt cascades (8, 13). We previously demonstrated that OPG drastically reduced caspase-3/7 activities and maintained ECFCs viability after 48 h of treatment. This was supported by an observed down-regulation of pro-apoptotic genes (Hyou1, Taldo1 (transaldolase), crk (adapter molecule crk), and Sh3glb1 (endotphilin-B1) in OPG-stimulated ECFCs that reflects the capacity of OPG to protect cells from apoptosis and to promote their survival.

### ECFCs priming of OPG induces overexpression of CD34^+^

ECFCs originate from the blood-derived mononuclear progenitor cells fraction expressing CD34 but during their *in vitro* endothelial expansion part of the cells loses CD34 upon differentiation. The function of this transmembrane cell surface glycoprotein remains obscure, though CD34 has been shown to be a ligand for L-selectin allowing leucocytes adhesion to the endothelium at sites of inflammation (15, 16). Our finding that OPG increases CD34 could reflect a mobilization of a more immature state of ECFCs. Previous study on CD34 in ECFCs suggests that CD34 expression is not related to different ECFCs subpopulations but is a reflect of cell plasticity and the result of angiogenic stimuli interfering with the endothelial tube formation (32). The CD34 expression is known to be inducible and down-regulated, in HUVECs, by angiogenic growth factors (32, 33). Thus, upregulation of CD34 observed in OPG-exposed ECFCs could be both attributed to progenitor subtype expansion but also to an increased ECFCs vasculogenic potential.

Our data obtained on cord blood-derived ECFCs need now to be confirmed with adult peripheral blood mobilized or not with G-CSF. This validation will allow us to propose OPG as a cell culture adjuvant for ECFCs isolation in GMP culture condition. Moreover, this potential link between OPG and endothelial differentiation could also be tested on stem cell at the origin of endothelial lineage. We recently described that very small embryonic-like stem cells (VSELs) were able to differentiate in endothelial lineage (34, 35). making these cells a potential cell source for cell therapy. VSELs exhibit several features of pluripotent stem cells and do not form teratomas after transplantation into deficient mice. As a prerequisite step to cell therapy trials, as an expansion model have just been described by Pr Ratajczak's group, OPG could be now used in new differentiation ongoing protocol.

In conclusion, this study provides further evidence that OPG plays a functional role in ECFCs commitment and expansion In addition to its action on angiogenesis, our work confirms its important contribution to the angiogenic/vasculogenic process. OPG secretion from vascular cells and platelets may be involved in blood-derived ECFCs maturation, proliferation, and their ability to form new blood vessels. It may provide new opportunities for optimization of *ex vivo* expansion and maintenance of human CD34^+^ progenitors such as ECFCs or VSELs in appropriate media free from feeder-layer cells, and may have value for their application in regenerative medicine. A better understanding of this OPG/CD34 pathway in vasculogenesis could lead to new strategies for *ex vivo* ECFCs expansion.

## Ethics statement

The study was approved both by the relevant ethics committee (Hôpital Saint Louis, Paris, France) and the French Ministry of Higher Education, Training and Scientific Research (AC-2008-376). The protocol complied with the Declaration of Helsinki. Umbilical cord blood were collected after normal full-term deliveries with the written informed consent of the mother, and used within 24 h.

## Author contributions

CB-V and ZB-A designed the *in vitro* experiments and performed data analysis. ZB-A and AL conducted the experiments, interpreted, and analyzed data. ZB-A and CB-V wrote the manuscript. DH and DS performed critical revising of the intellectual content. CB-V and DH financially supported the experiments. All authors read and approved the final manuscript.

### Conflict of interest statement

The authors declare that the research was conducted in the absence of any commercial or financial relationships that could be construed as a potential conflict of interest.
